# Combination therapy with 3-Hydroxybenzaldehyde and Albendazole modulates mitochondrial protein expression in astrocytes after *Angiostrongylus cantonensis* infection

**DOI:** 10.1371/journal.pntd.0013773

**Published:** 2025-11-24

**Authors:** Yi-Hao Huang, Shih-Hsin Chang, Chien-Ju Cheng, Yi-Hsuan Lin, Yu-Chi Chou, Chyi-Liang Chen, Kuang-Yao Chen

**Affiliations:** 1 Graduate Institute of Biomedical Sciences, College of Medicine, Chang Gung University, Taoyuan, Taiwan; 2 School of Medicine, College of Medicine, MacKay Medical University, New Taipei, Taiwan; 3 Department of Parasitology, College of Medicine, Chang Gung University, Taoyuan, Taiwan; 4 Molecular Infectious Disease Research Center, Chang Gung Memorial Hospital, Taoyuan, Taiwan; University of Buea, CAMEROON

## Abstract

**Background:**

*Angiostrongylus cantonensis* is a zoonotic nematode that causes eosinophilic meningitis and central nervous system injury in humans; 3-hydroxybenzaldehyde (3-HBA) is a benzaldehyde compound that exhibits antioxidant and anti-inflammatory activities. Brain injury promotes Ca²⁺ influx and mitochondrial Ca²⁺ loading via voltage-dependent anion channel 1 (VDAC1) and the mitochondrial Ca²⁺ uniporter (MCU), leading to mitochondrial dysfunction and cytochrome c-mediated apoptosis.

**Methodology/Principal Findings:**

This study aimed to evaluate the therapeutic effects of 3-HBA combined with albendazole on brain injury and the expression of mitochondria-related molecules in *A. cantonensis*-infected mice. In BALB/c mice infected with *A. cantonensis*, the infection significantly increased glial fibrillary acidic protein expression in five regions: the cerebral cortex, hippocampus, subcortical areas, cerebellum, and brainstem and elevated the expression of MCU and cytochrome c in the cerebral cortex and hippocampus. Hematoxylin and eosin staining revealed pathological changes, such as meningitis, hemorrhage, and vascular congestion. However, combined treatment with 3-HBA and albendazole reduced these pathological changes and the expression of mitochondria-related molecules, including glial fibrillary acidic protein, VDAC1, MCU, and cytochrome c. In cultured mouse astrocytes, soluble antigens from fifth-stage larval-activated astrocytes induced mitochondria-related molecule expression, but 3-HBA suppressed these effects.

**Conclusions/Significance:**

These results suggest that the combination of 3-HBA and albendazole downregulates astrocyte activation and VDAC1/MCU-associated mitochondrial pathways following *A. cantonensis* infection. These findings support the use of 3-HBA as a promising adjuvant to albendazole in the treatment of angiostrongyliasis.

## Author summary

*Angiostrongylus cantonensis* (rat lungworm) is a major food-borne zoonotic parasite that causes severe central nervous system pathologies in humans, including eosinophilic meningitis and meningoencephalitis. Therapeutic strategies for cerebral angiostrongyliasis remain controversial; 3-hydroxybenzaldehyde (3-HBA) is a benzaldehyde derivative. Our previous studies demonstrated that treatment with 3-HBA combined with an anthelmintic exerted therapeutic effect by modulating oxidative stress and apoptosis in *A. cantonensis*-infected mouse brains. Therefore, this study aimed to elucidate the mitochondrial mechanisms underlying these effects. We investigated the mechanism by which 3-HBA regulates mitochondria-associated pathways during *A. cantonensis* infection. These findings enhance our understanding of 3-HBA-mediated neuroprotection and may inform the development of adjunctive therapeutic strategies for human angiostrongyliasis.

## Introduction

*Angiostrongylus cantonensis*, commonly known as the rat lungworm, is a parasitic nematode that causes angiostrongyliasis in humans. This zoonotic disease can lead to conditions, such as eosinophilic meningitis and meningoencephalitis, which may result in severe or even fatal outcomes. The optimal treatment for cerebral angiostrongyliasis remains a topic of ongoing debate [1]. The life cycle of *A. cantonensis* includes rats as definitive hosts and mollusks as intermediate hosts. Adult worms reside and reproduce in the pulmonary arteries and right ventricles of rats. Female worms lay eggs that hatch into first-stage larvae within the blood capillaries of the lungs. These larvae are eventually expelled in rat feces. Mollusks become infected either by ingesting larvae or through skin penetration. Humans, as accidental hosts, become infected when they consume an intermediate or paratenic host containing infective third-stage (L3) larvae. The larvae penetrate the intestinal wall, enter the bloodstream, migrate to the central nervous system (CNS), and develop into fifth-stage (L5) larvae [2].

Infection with L5 larvae can cause severe inflammatory responses, mechanical injury, and cell death, particularly in humans. This process involves the recruitment of eosinophils and secretion of cytokines and chemokines within the CNS [3–8]. In our study of *A. cantonensis*, we found that oxidative stress, apoptosis, and inflammation were induced in the brains of infected mice [5,9].

Astrocytes are the most abundant glial cells in the CNS and interact with various brain cell types to perform different functions, such as maintaining neurotransmitter homeostasis, supporting synapse formation and plasticity, clearing excess neurotransmitters, and contributing to the formation of the blood–brain barrier [10,11]. Astrocytes play a critical role in CNS disorders and neurodegenerative diseases, including autoimmune inflammation, multiple sclerosis, and Alzheimer’s disease [12].

Furthermore, mitochondrial dysfunction in astrocytes is a key contributor to the pathology of neurodegenerative diseases [13,14]. In Alzheimer’s disease, mitochondrial dysfunction has been observed, including reduced mitochondrial metabolic capacity, decreased mtDNA synthesis, increased reactive oxygen species (ROS) production, and impaired lactate generation [15].

3-Hydroxybenzaldehyde (3-HBA) is an oxidized benzaldehyde with a hydroxyl group in its structural unit and possesses antioxidant, anti-inflammatory, and antimicrobial properties [1,16,17]. 3-HBA has vasoprotective potential, which suggests that it could be used in the treatment of atherosclerosis [16]. Additionally, 3-HBA has been evaluated as an experimental compound for new cancer treatments [18]. Our previous research showed that combined treatment with albendazole and 3-HBA in *A. cantonensis*-infected mice enhanced antioxidant activity, reduced the expression of apoptosis-related molecules in the brain, and increased cell viability [1,19]. Moreover, 3-HBA inhibits the production of ROS, thereby reducing endoplasmic reticulum stress [20].

The infection with *A. cantonensis* in mice induces oxidative stress in the CNS, ultimately leading to astrocyte apoptosis and brain injury. By contrast, mitochondrial dysfunction is recognized as a key mechanism through which astrocytes contribute to the pathogenesis of neurodegenerative diseases. Therefore, in this study, we investigated the therapeutic effects of 3-HBA combined with albendazole against *A. cantonensis*-induced pathogenesis, focusing on mitochondrial function. The results of this study elucidate the therapeutic capacity of 3-HBA as an adjuvant therapeutic agent and provide novel insights into potential treatment strategies for CNS injury caused by parasitic infections.

## Materials and methods

### Ethical approval

All animal procedures were reviewed and approved by the Institutional Animal Care and Use Committee of Chang Gung University, Taiwan (IACUC approval CGU111–167 and CGU113–082), and conducted in accordance with the Guidelines for Laboratory Animal Facilities and Care issued by the Council of Agriculture, Executive Yuan (ROC). Rats and mice were housed in plastic cages with ad libitum access to food and water. Animals were euthanized under inhalational isoflurane anesthesia (1 mL/min).

### Establishment and maintenance of *A. cantonensis* life cycle

The *A. cantonensis* strain used in this study was originally isolated from *Achatina fulica* in Neihu, Taipei, in 1985. The laboratory life cycle was established by infecting Sprague–Dawley rats with L3 larvae [2]. First-stage larvae were collected from rat feces using a modified Baermann funnel method [21] and used to infect *Biomphalaria glabrata*. After 21 d, L3 larvae were harvested from the infected snails by tissue homogenization (Cole-Parmer Instrument Co., USA), followed by enzymatic digestion with artificial gastric juice (0.6% w/v pepsin, pH 2–3). L3 larvae were then used to infect rats by oral inoculation.

### Experimental animals

Sprague–Dawley rats and BALB/c mice were purchased from the National Laboratory Animal Center, Taipei, Taiwan, for the maintenance of the *A. cantonensis* life cycle. Animals were housed individually in plastic cages under controlled conditions with free access to standard chow and drinking water. All procedures involving animal care and handling were conducted at the Chang Gung University Animal Center and reviewed and approved by the Institutional Animal Care and Use Committee of Chang Gung University.

### Drug treatment

In this study, BALB/c mice were infected orally with 25 L3 larvae of *A. cantonensis*. Body weight was monitored daily throughout the experimental period. After 7 d, the mice received albendazole (10 mg/kg) and 3-HBA (100 mg/kg) once daily until they were sacrificed at 21 dpi. At 21 dpi, the mice were euthanized, and blood and brain tissues were collected. The brains were dissected into five regions: the cerebral cortex, hippocampus, subcortical areas, cerebellum, and brainstem.

### Immunohistochemistry staining

Brain tissues were processed for paraffin embedding. Samples were fixed in 10% formalin and dehydrated in a graded ethanol series. The dehydrated tissues were cleared in xylene for 1 h to ensure transparency and compatibility with paraffin infiltration. Subsequently, the brain sections were washed with PBS. H&E staining was performed to evaluate the pathological feature. Finally, the slides were dehydrated in xylene and permanently mounted using NeoMount (Cat# 1.09016; Merck, Germany). Images were acquired using a light microscope.

### Hematoxylin and eosin (H&E) staining and histopathological assessment

H&E staining was performed to evaluate four pathological features: meningitis, congestion, hemorrhage, and the presence of larvae. The evaluation criteria were adapted from previously established methods [22,23].

### Western blot analysis

Protein samples were separated on 12% sodium dodecyl sulfate–polyacrylamide gels and transferred onto nitrocellulose membranes using a wet-transfer system. Membranes were blocked in blocking buffer before incubation with primary antibodies overnight at 4 °C. The following primary antibodies were used: GFAP (Proteintech, USA), VDAC1 (ABclonal, USA), MCU (ABclonal, USA), cytochrome c (ABclonal, USA), and β-actin (Sigma-Aldrich, USA). The membranes were then incubated with horseradish peroxidase-conjugated secondary antibodies. The blots were visualized using enhanced chemiluminescence with equal volumes of stable peroxide and luminol enhancer solutions.

### Cell culture

Mouse astrocytes (CRL-2535) were obtained from the American Type Culture Collection (Manassas, VA, USA). The cells were maintained in Dulbecco’s modified Eagle’s medium (Corning, NY, USA) supplemented with 10% fetal bovine serum and 100 U/ml penicillin–streptomycin. Cells were seeded at a density of 0.25 × 10^6^ cells/cm2 onto poly-L-lysine-coated culture plates and incubated at 37 °C in 5% CO₂ until they reached confluence (1–2 × 10^4^ cells/cm2). Immunostaining for GFAP confirmed that >95% of the cultured cells were astrocytes.

### Immunofluorescence staining

Cells were fixed with 3.7% paraformaldehyde and treated with 0.1% Triton X-100. After blocking with 1% BSA, the cells were incubated with primary antibodies (GFAP, Proteintech, USA; VDAC1, ABclonal, USA; MCU, ABclonal, USA; cytochrome c, ABclonal, USA), followed by incubation with a fluorescent secondary antibody. Nuclei were counterstained with DAPI using the mounting medium.

### Statistical analysis

All data were analyzed using GraphPad Prism 8.0 software. Statistical significance was determined using a one-way ANOVA or Student’s t-test. Data are expressed as mean ± SD. p < 0.05, p < 0.01, p < 0.001, and p < 0.0001 were considered statistically significant.

## Results

### Evaluation of body weight changes in BALB/c mice infected with *A. cantonensis* and therapeutic treatment

To evaluate the therapeutic effect of 3-HBA in *A. cantonensis*-infected mice, we monitored body weight following L3 larvae infection and treatment. The animals were infected with 25 L3 larvae and assigned to four groups (n = 13/group): normal (uninfected), infected, infected + albendazole, and infected + albendazole + 3-HBA. Body weight was recorded daily, and treatments were initiated from 7 to 21 dpi.

**Fig 1:**
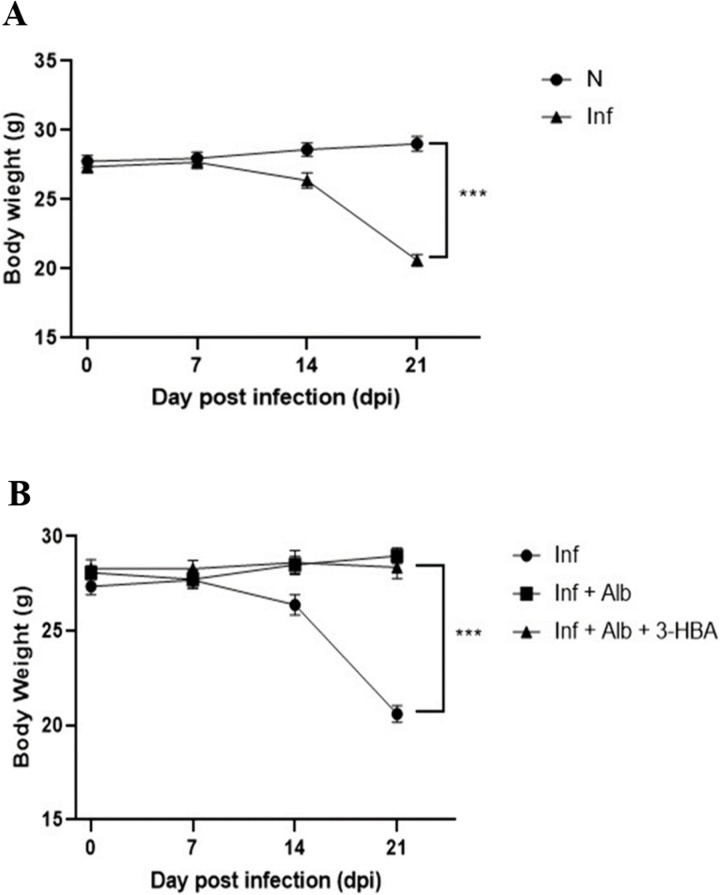
Evaluation of the body weight changes in BALB/c mice after *Angiostrongylus cantonensis* infection or therapeutic treatment. Mice were assigned to four groups (*n* = 11/group): Normal **(N)**, Infected (Infect), Infected + albendazole (Infect + Alb), and Infected + albendazole + 3-HBA (Infect + Alb + 3-HBA). Body weight was recorded daily for 21 days. **(A)** The changes in body weight for N and Infect groups at 0, 7, 14, and 21 days post-infection (dpi). **(B)** The changes in body weight for Infect, Infect + Alb, and Infect + Alb + 3-HBA groups at 0, 7, 14, and 21 dpi. The data are obtained from independent experiments; ***P < 0.001.

As shown in Fig 1A, the body weight of the infected group significantly decreased by day 21. Moreover, on day 21, the infected + albendazole and infected + albendazole + 3-HBA groups exhibited significantly higher body weights than those of the infected group (Fig 1B). These data indicate that 3-HBA combined with albendazole is an effective therapeutic strategy against *A. cantonensis* infection.

### Evaluation of pathological changes in BALB/c mice infected with *A. cantonensis* and therapeutic treatment

**Fig 2:**
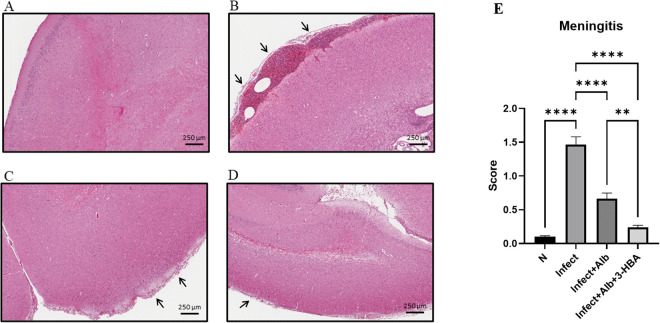
Evaluation of the effect of 3-HBA on eosinophilic meningitis in BALB/c mice after *A. cantonensis* infection. H&E stained brain tissues from **(A)** Normal **(N)**, **(B)** Infected (Infect), **(C)** Infected + albendazole (Infect + Alb), and **(D)** Infected + albendazole + 3-HBA (Infect + Alb + 3-HBA) **(E)** Quantification of eosinophilic meningitis thickness. The data are obtained from independent experiments (*n* = 3); **P < 0.01, ****P < 0.0001.

**Fig 3:**
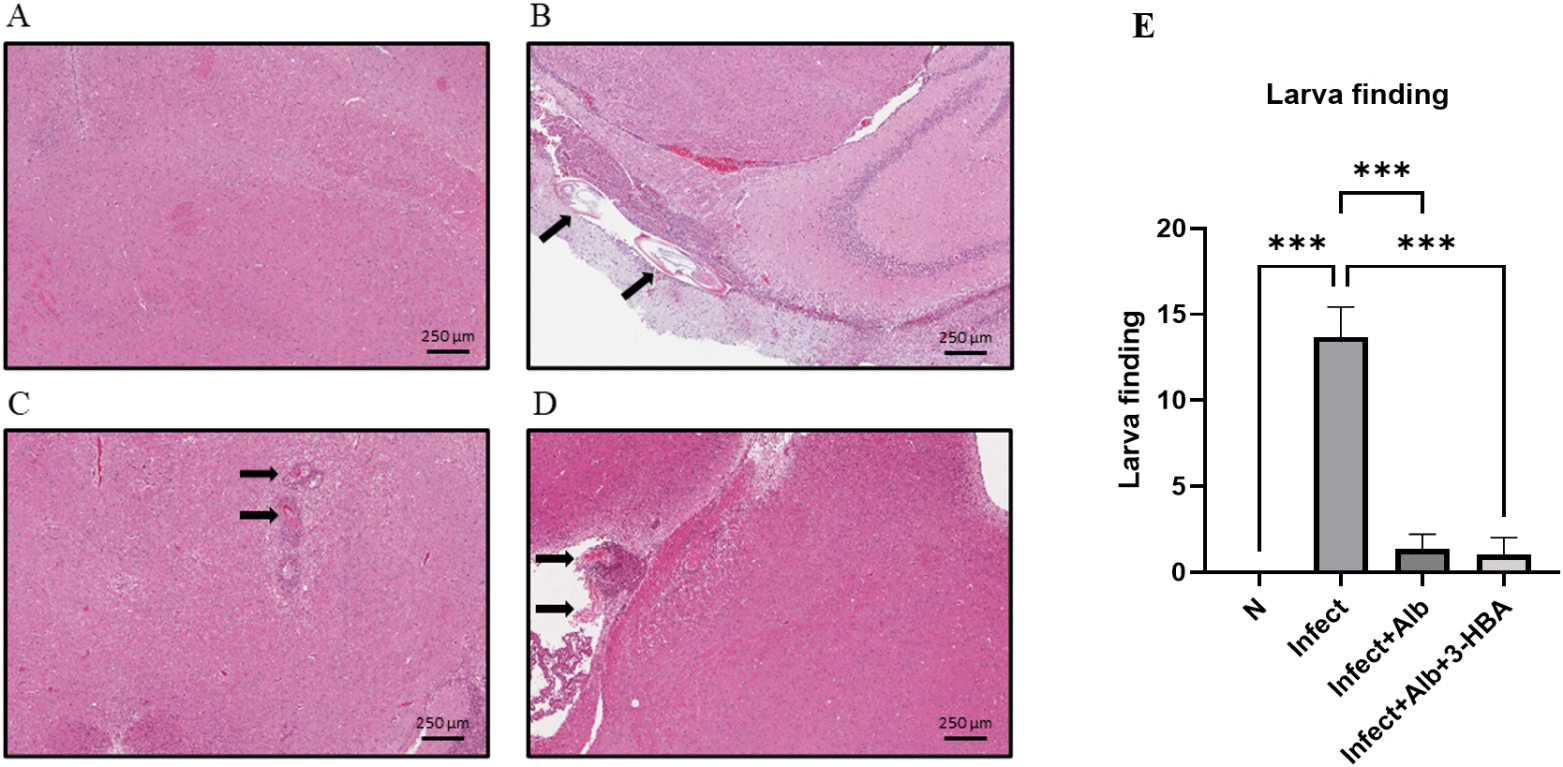
Evaluation of the effect of 3-HBA on larval presence in BALB/c mice after *A. cantonensis* infection. H&E stained brain tissues from **(A)** Normal **(N)**, (B) nfected (Infect), **(C)** Infected + albendazole (Infect + Alb), and **(D)** Infected + albendazole + 3-HBA (Infect + Alb + 3-HBA) **(E)** Quantification of larval presence. The data are obtained from independent experiments (*n* = 3); ***P < 0.001.

**Fig 4:**
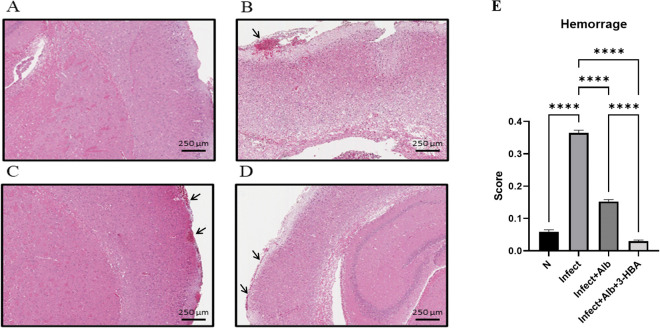
Evaluation of the effect of 3-HBA on hemorrhage in BALB/c mice after *A. cantonensis* infection. H&E stained brain tissues from **(A)** Normal **(N)**, (B) nfected (Infect), **(C)** Infected + albendazole (Infect + Alb), and **(D)** Infected + albendazole + 3-HBA (Infect + Alb + 3-HBA) **(E)** Quantification of hemorrhage. The data are obtained from independent experiments (*n* = 3); ****P < 0.0001.

**Fig 5:**
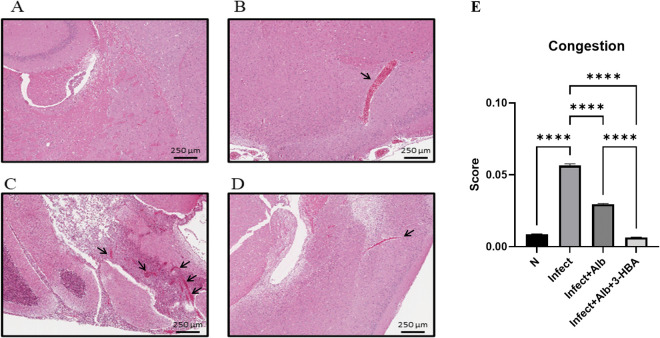
Evaluation of the effect of 3-HBA on vascular congestion in BALB/c mice after *A. cantonensis* infection. H&E stained brain tissues from **(A)** Normal **(N)**, (B) nfected (Infect), **(C)** Infected + albendazole (Infect + Alb), and **(D)** Infected + albendazole + 3-HBA (Infect + Alb + 3-HBA) **(E)** Quantification of vascular congestion. The data are obtained from independent experiments (*n* = 3); ****P < 0.0001.

To evaluate the therapeutic effect of 3-HBA on pathological changes in the CNS after *A. cantonensis* infection, histopathological examination was performed using H&E staining. We detected four features: eosinophilic meningitis, larval findings, hemorrhage, and vascular congestion. As shown in Figs 2–5, these pathological features were significantly increased in the infected group. However, the combination of albendazole and 3-HBA significantly reduced pathological damage. Collectively, these findings indicate that 3-HBA attenuates severe neuropathological changes in the brains of mice following *A. cantonensis* infection.

### Effect of astrocyte activation following treatment with 3-HBA in BALB/c mice infected with *A. cantonensis*

**Fig 6:**
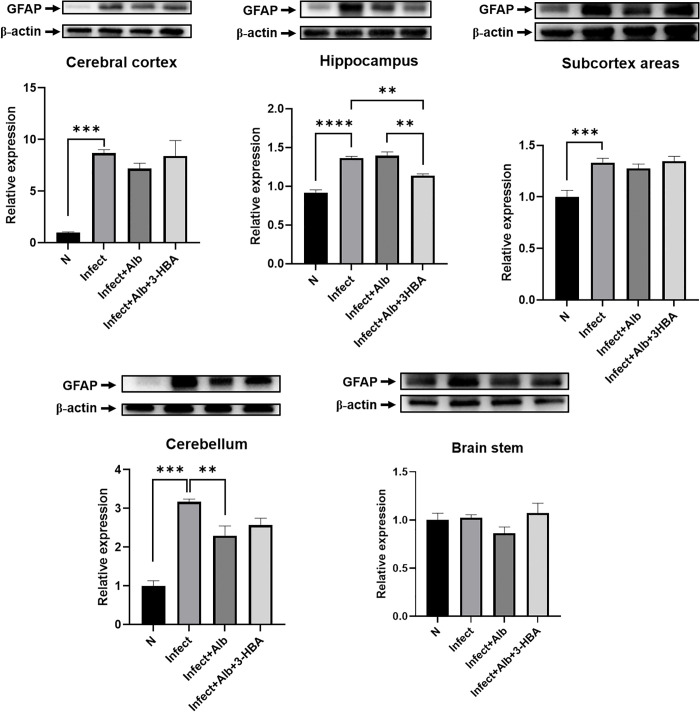
The protein expression of GFAP in *A. cantonensis*-infected mice after albendazole and 3-HBA treatment. The western blotting was employed to detect the protein expression of GFAP in each treatment group *in vivo.* (A) cerebral cortex, (B) hippocampus, (C) subcortical areas, (D) cerebellum, and (E) brain stem. N: Normal, Inf: Infection, Inf + Alb: infection+albendazole, Inf + Alb + 3-HBA: Infection+albendazole+3-HBA. The data are expressed as the means ± SEM from independent experiments (*n* = 3); *P < 0.05, **P < 0.01, ***P < 0.001.

First, we determined whether 3-HBA could reduce astrocyte activation in *A. cantonensis*-infected BALB/c mice. Western blotting was used to detect the protein expression of GFAP in five brain regions: the cerebral cortex, hippocampus, subcortex, cerebellum, and brainstem. As shown in Fig 6, GFAP expression was significantly elevated in the cerebral cortex, hippocampus, subcortex, and cerebellum after *A. cantonensis* infection. Albendazole combined with 3-HBA significantly downregulated GFAP expression in the hippocampus. These findings demonstrate that 3-HBA attenuates astrocyte activation in BALB/c mice following *A. cantonensis* infection, with particularly pronounced effects in the hippocampus.

### Evaluation of VDAC1 expression following treatment with 3-HBA in BALB/c mice infected with *A. cantonensis*

**Fig 7:**
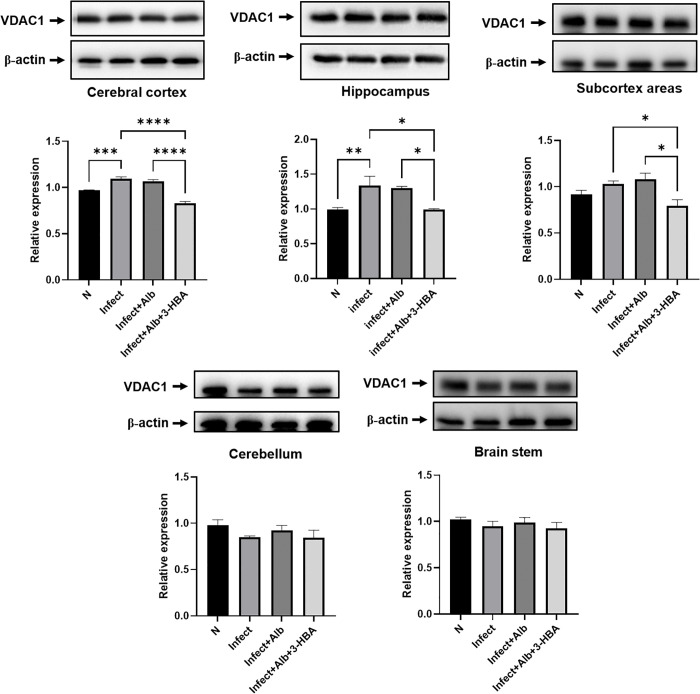
The protein expression of VDAC1 in *A. cantonensis*-infected mice after albendazole and 3-HBA treatment. The western blotting was employed to detect the protein expression of VDAC1 in each treatment group *in vivo.* (A) cerebral cortex, (B) hippocampus, (C) subcortical areas, (D) cerebellum, and (E) brain stem. N: Normal, Inf: Infection, Inf + Alb: infection+albendazole, Inf + Alb + 3-HBA: Infection+albendazole+3-HBA. The data are expressed as the means ± SEM from independent experiments (*n* = 3); *P < 0.05, **P < 0.01, ***P < 0.001, ****P < 0.0001.

During the initiation of mitochondria-mediated intrinsic apoptosis, VDAC1 expression increases, promoting the release of cytochrome c into the cytosol and triggering downstream apoptotic cascades [24]. As shown in Fig 7, VDAC1 expression was significantly elevated in the cerebral cortex and hippocampus after *A. cantonensis* infection. In addition, albendazole combined with 3-HBA significantly downregulated VDAC1 expression in the cerebral cortex, hippocampus, and subcortex. These findings demonstrate that 3-HBA downregulates VDAC1 expression in brain regions following *A. cantonensis* infection.

### Evaluation of MCU expression following treatment with 3-HBA in BALB/c mice infected with *A. cantonensis*

**Fig 8:**
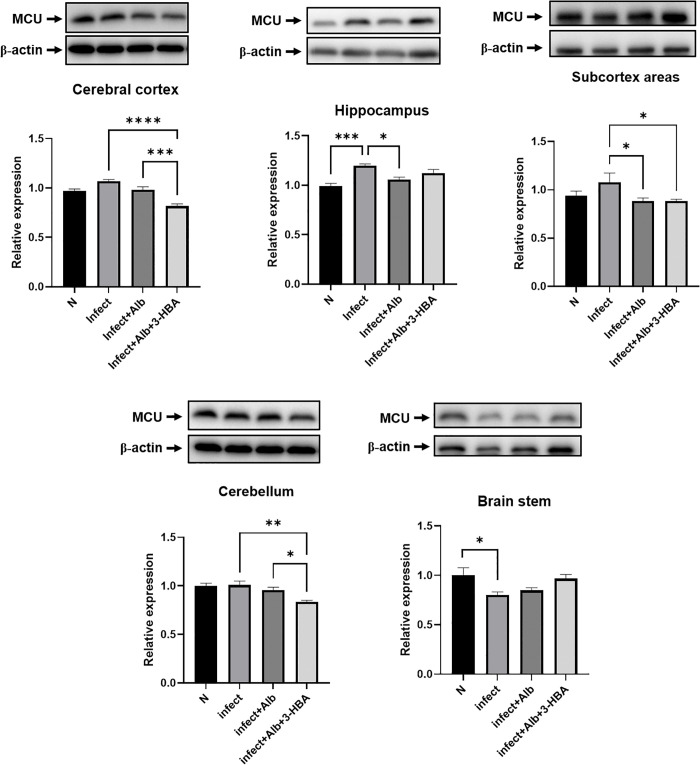
The protein expression of MCU in *A. cantonensis*-infected mice after albendazole and 3-HBA treatment. The western blotting was employed to detect the protein expression of MCU in each treatment group *in vivo.* (A) cerebral cortex, (B) hippocampus, (C) subcortical areas, (D) cerebellum, and (E) brain stem. N: Normal, Inf: Infection, Inf + Alb: infection+albendazole, Inf + Alb + 3-HBA: Infection+albendazole+3-HBA. The data are expressed as the means ± SEM from independent experiments (*n* = 3); *P < 0.05, **P < 0.01, ***P < 0.001, ****P < 0.0001.

Under cellular injury conditions, Ca2+ is transported into the mitochondrial matrix through the MCU. Excessive mitochondrial Ca2+ loading increases ROS levels, disrupts mitochondrial function, and stimulates the intrinsic apoptotic pathway [25]. As shown in Fig 8, MCU expression was significantly elevated in the hippocampus after *A. cantonensis* infection. In addition, albendazole combined with 3-HBA significantly downregulated MCU expression in the cerebral cortex, subcortex, and cerebellum. These findings demonstrate that 3-HBA downregulates MCU expression in various brain regions following *A. cantonensis* infection.

### Evaluation of cytochrome c expression following treatment with 3-HBA in BALB/c mice infected with *A. cantonensis*

**Fig 9:**
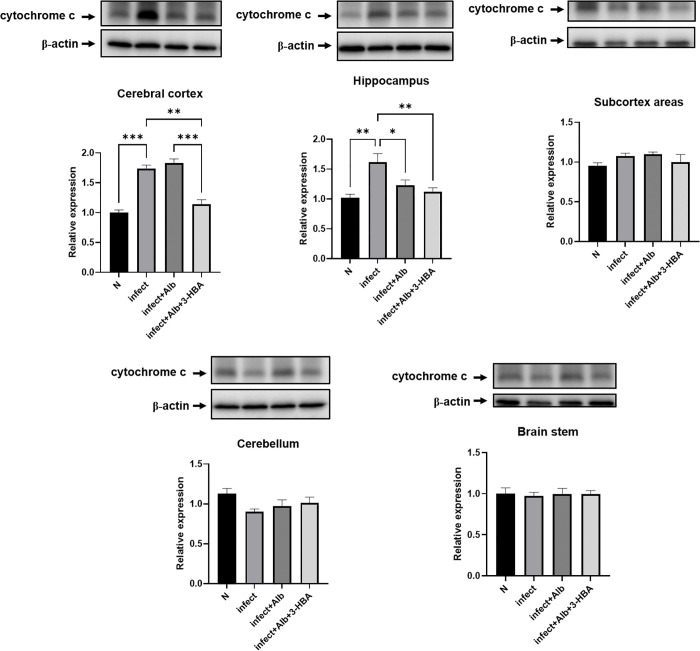
The protein expression of cytochrome c in *A. cantonensis*-infected mice after albendazole and 3-HBA treatment. The western blotting was employed to detect the protein expression of cytochrome c in each treatment group *in vivo.* (A) cerebral cortex, (B) hippocampus, (C) subcortical areas, (D) cerebellum, and (E) brain stem. N: Normal, Inf: Infection, Inf + Alb: infection+albendazole, Inf + Alb + 3-HBA: Infection+albendazole+3-HBA. The data are expressed as the means ± SEM from independent experiments (*n* = 3); *P < 0.05, **P < 0.01, ***P < 0.001.

Mitochondrial damage alters membrane permeability, leading to the release of cytochrome c into the cytosol and initiation of apoptosis. As shown in Fig 9, cytochrome c expression was significantly elevated in the cerebral cortex and hippocampus after *A. cantonensis* infection. Additionally, albendazole combined with 3-HBA significantly downregulated cytochrome c expression in these two regions. These findings demonstrate that 3-HBA downregulates cytochrome c expression in BALB/c mice following *A. cantonensis* infection, particularly in the cerebral cortex and hippocampus.

### Expression of GFAP and mitochondria-related proteins in astrocytes following exposure to *A. cantonensis* L5 soluble antigens (SA)

**Fig 10:**
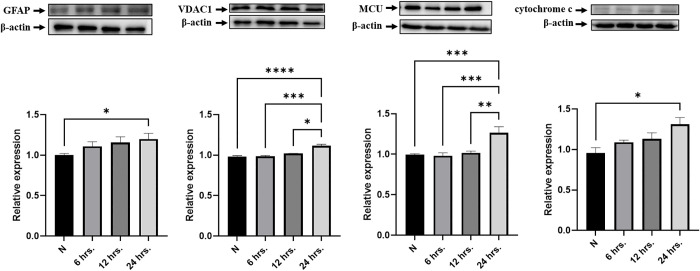
The protein expression of GFAP and mitochondria-related proteins in *A. cantonensis* L5 soluble antigen stimulated astrocytes. Mouse astrocytes were treated with *A. cantonensis* L5 soluble antigen (SA) for 0 **(N)**, 6, 12, or 24 **h.** The western blotting was employed to detect the protein expression in each treatment group *in vitro.* **(A)** GFAP, **(B)** VDAC1, **(C)** MCU, and (D) cytochrome **c.** The data are expressed as the means ± SEM from independent experiments (*n* = 3); *P < 0.05, **P < 0.01, ***P < 0.001.

To evaluate the effects of *A. cantonensis* on the expression of mitochondria-related proteins (VDAC1, MCU, and cytochrome c) in astrocytes, astrocytes were stimulated with SA from *A. cantonensis* L5. As shown in Fig 10, the expression of these proteins was significantly elevated after SA treatment compared with that in the untreated group. These data indicate that *A. cantonensis* L5 SA stimulates astrocyte activation and induces mitochondria-related protein expression.

### 3-HBA downregulates mitochondria-related protein expression in astrocytes after *A. cantonensis* L5 SA treatment

**Fig 11:**
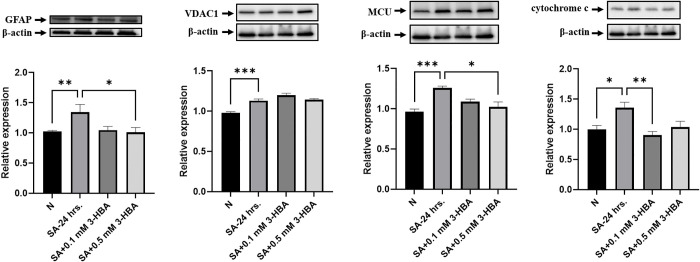
The effects of 3-HBA on GFAP and mitochondria-related proteins expression in *A.* *cantonensis* L5 soluble antigen stimulated astrocytes. Astrocytes were stimulated with L5 *A. cantonensis* L5 soluble antigen (SA) or 3-HBA (0.1 or 0.5 mM) for 24 h. The western blotting was employed to detect the protein expression in each treatment group *in vitro.* (A) GFAP, (B) VDAC1, (C) MCU, and (D) cytochrome c. The data are expressed as the means ± SEM from independent experiments (*n* = 3); *P < 0.05, ***P < 0.001, ****P < 0.0001.

**Fig 12:**
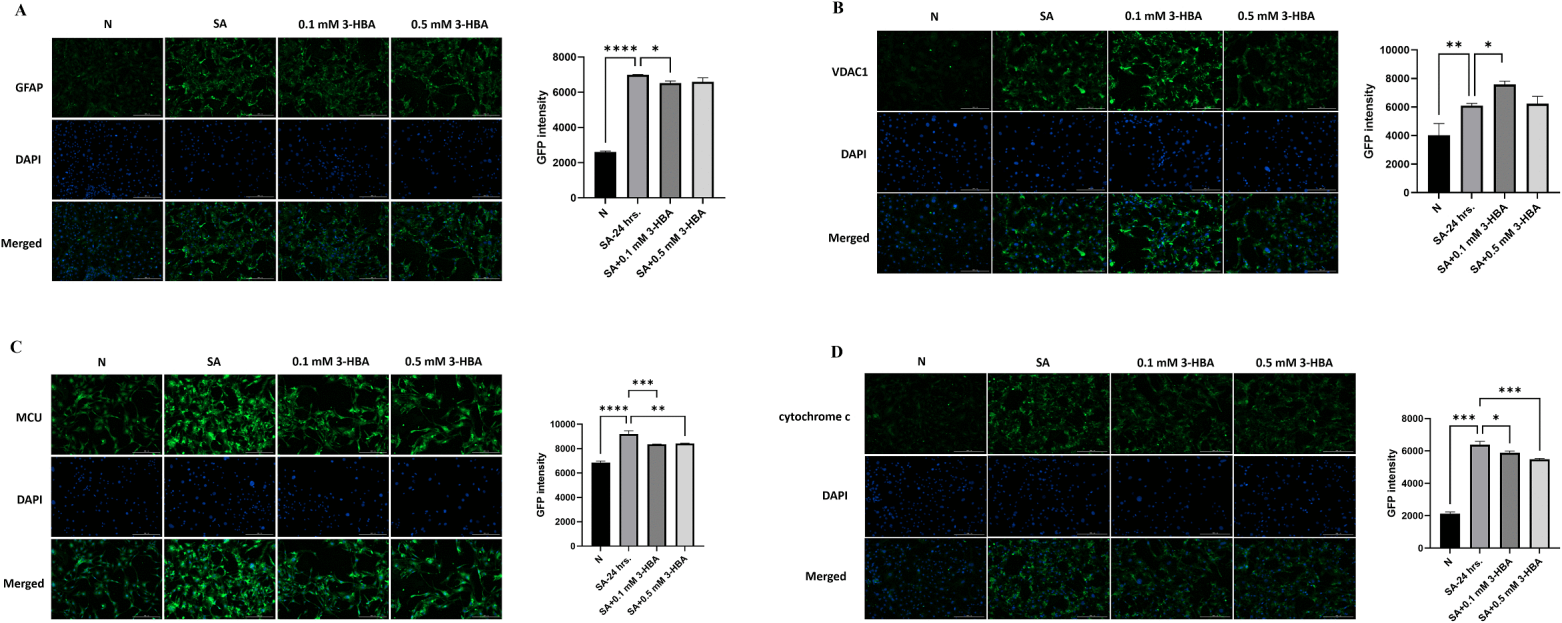
Immunofluorescence analysis of GFAP and mitochondria-related proteins after 3-HBA treatment in *A. cantonensis* L5 soluble antigen stimulated astrocytes. Astrocytes were stimulated with L5 *A. cantonensis* L5 soluble antigen (SA) or 3-HBA (0.1 or 0.5 mM) for 24 **h.** The immunofluorescence staining was employed to detect the protein expression in each treatment group *in vitro.* **(A)** GFAP, **(B)** VDAC1, **(C)** MCU, and (D) cytochrome **c.** The data are expressed as the means ± SEM from independent experiments (*n* = 5); *P < 0.05, **P < 0.01, ***P < 0.001.

We confirmed the effects of 3-HBA on the expression of mitochondria-related proteins (GFAP, VDAC1, MCU, and cytochrome c) in astrocytes. As shown in Fig 11, *A. cantonensis* L5 SA induced the expression of these proteins in astrocytes. Moreover, the expression of GFAP and cytochrome c was significantly decreased after 0.1 or 0.5 mM 3-HBA treatment compared with observations in SA alone. Immunofluorescence staining was then used to detect mitochondria-related proteins (GFAP, VDAC1, MCU, and cytochrome c) in astrocytes (Fig 12). The data showed that the expression of mitochondria-related proteins significantly increased after SA treatment but significantly decreased after 3-HBA treatment compared with that after SA alone. Collectively, these data demonstrate that 3-HBA attenuates astrocyte activation and reduces mitochondria-associated damage.

## Discussion

In this study, we evaluated the therapeutic potential of 3-HBA in angiostrongyliasis. We characterized CNS injury during *A. cantonensis* infection and assessed the protective efficacy of 3-HBA in combination with albendazole. In addition, we evaluated the therapeutic effects of 3-HBA on astrocytic responses after stimulation with SA derived from *A. cantonensis* L5 larvae. *A. cantonensis* infection induces mitochondrial injury in the CNS [26]. Therefore, we primarily focused on investigating mitochondria-related pathways in astrocytes.

3-HBA is a relatively small molecular weight, lipophilic phenolic compound (a phenolic aldehyde contains one hydroxyl group and one aldehyde group), and a few polar substituents. It may have the capacity to facilitate passive diffusion across the blood-brain barrier (BBB). Moreover, previous studies on *A. cantonensis* infection have demonstrated that the BBB can be disrupted or become leaky to some degree, leading to clinical signs such as neuroinflammation or hemorrhage. This BBB breakdown could facilitate the enhanced passage of compounds that would otherwise be restricted. Our previous investigation also clarified the protective efficacy of 3-HBA in the mouse brain after *A. cantonensis* infection. We showed that 3-HBA exhibits BBB protective efficacy in the CNS [1]. In conclusion, 3-HBA (alone or in combination with albendazole) could reach the CNS under infection conditions. However, this characteristic requires further experimental confirmation, such as using a radiolabeled tracer. Investigating the therapeutic efficacy of 3-HBA in the CNS will help us further understand its potential for the treatment of neuroangiostrongyliasis.

Our previous studies demonstrated the therapeutic potential of 3-HBA in the *A. cantonensis*-infected mouse brain [1,19,20]. The data revealed that 3-HBA exhibits potent antioxidant and anti-inflammatory properties, which are closely linked to its ability to inhibit cell apoptosis in astrocytes and prevent breakdown of the BBB. After *A. cantonensis* infection, the neuroinflammation and cell apoptosis can stimulate CNS pathology changes and neuronal injury. 3-HBA treatment was shown to reduce oxidative stress and astrocytic hyperactivation, thereby improving BBB function. However, treatment with 3-HBA alone did not demonstrate direct parasiticidal activity against *A. cantonensis* L5. This compound role is predominantly host-directed, aiming to counteract infection-induced oxidative and inflammatory damage in the CNS. In combination therapy, 3-HBA combined with albendazole showed synergistic benefits (albendazole exerted an anthelmintic effect, whereas 3-HBA provided neuroprotective and anti-inflammatory actions). Therefore, while 3-HBA does not directly kill the *A. cantonensis* L5, it acts as a neuroprotective adjunct that enhances CNS recovery and reduces neuroinflammation after *A. cantonensis* infection. These data revealed that 3-HBA is a potential therapeutic candidate for use alongside conventional anthelmintics.

The MCU is the principal Ca2+ uptake channel in the inner mitochondrial membrane. LPS activates the IP3R–GRP75–VDAC1–MCU Ca2+ transfer axis, thereby inducing Ca2+ entry into the mitochondrial matrix [27]. Moreover, the MCU contributes to neurotransmission and redox homeostasis in the CNS [28]. The MCU has also been implicated in hippocampal neuroinflammation in Parkinson’s disease [29] and in synaptic loss following brain injury [30]. In this study, we observed that astrocyte activation (GFAP expression) and MCU expression were significantly elevated in the hippocampus after *A. cantonensis* infection *in vivo* and after L5 larvae SA treatment *in vitro*. These findings support the hypothesis that *A. cantonensis* infection enhances mitochondrial Ca2+ uptake by activated astrocytes. Our previous work showed that 3-HBA has antioxidant capacity *in vitro* [20]. In the present study, 3-HBA combined with albendazole reduced MCU expression in the cerebral cortex, subcortex, and cerebellum *in vivo*.

Cytochrome c is a component of the electron transport chain that plays a major role in redox regulation and apoptosis. In CNS injury, excessive Ca2+ influx causes mitochondrial dysfunction, oxidative stress, and cell death [31]. In cerebral ischemia, mitochondrial Ca2+ accumulation and cytochrome c release trigger caspase-dependent apoptosis [32]. Our previous data confirmed that *A. cantonensis* infection or ESP stimulation elevated intracellular Ca2+ in the hippocampus [33]. In the present study, we found that cytochrome c expression significantly increased in astrocytes. These data suggest that *A. cantonensis* infection stimulates mitochondrial Ca2+ overload and ROS production, thereby promoting cytochrome c release and apoptosis. However, 3-HBA attenuates this mechanism [1,19].

Regional variation in mitochondrial protein expression likely reflects the distinct metabolic demands and vulnerability of different brain regions during *A. cantonensis* infection. In our observations, the cortex exhibited a more pronounced decrease in mitochondrial proteins, such as VDAC1 and MCU, accompanied by reduced retention of cytochrome C. In contrast, the cerebellum maintained relatively stable mitochondrial protein expression and lower ROS accumulation, suggesting an intrinsic resistance or delayed response to infection-induced stress.

3-hydroxybenzaldehyde (3-HBA) combined with albendazole treatment can restore mitochondrial function through several complementary mechanisms. Our previous studies determined that 3-HBA can enhance antioxidant capacity via reducing reactive oxygen species and improving antioxidant activity, thereby supporting mitochondrial integrity and energy metabolism in astrocytes. Concurrently, albendazole can eliminate *A. cantonensis* L5, alleviating ongoing inflammatory stress that suppresses mitochondrial biogenesis. Although this investigation has not yet directly quantified markers of mitochondrial biogenesis, such as TFAM, the observed astrocytic mitochondrial protein expression suggests that 3-HBA combined with albendazole therapy may promote mitochondrial recovery at both functional and structural levels. This possibility should be discussed as a future direction, emphasizing the potential of host-targeted metabolic restoration as an adjunct strategy for antiparasitic therapy in neuroangiostrongyliasis.

Subsequently, we examined the neuropathology of *A. cantonensis*-infected mice. Mitochondrial repair reduces oxidative stress, apoptosis, neuroinflammation, and astrocyte reactivity in traumatic brain injury [31]. Mitochondrial damage promotes inflammation by releasing mtDNA and other DAMPs that activate PRRs and inflammasomes [34]. Moreover, our investigations showed that *A. cantonensis* induces meningitis, hemorrhage, and vascular congestion *in vivo* and upregulates IL-1β and IL-6 secretion following ESP stimulation in astrocytes *in vitro* [1,5]. In the present study, H&E staining confirmed that meningitis, hemorrhage, and congestion increased after infection and were markedly ameliorated by combined 3-HBA and albendazole therapy.

In summary, we determined that *A. cantonensis* triggers mitochondrial dysfunction in astrocytes both *in vitro* and *in vivo*. However, 3-HBA attenuates this injury by reducing astrocyte activation and the expression of mitochondria-related molecules. Based on our findings, a combination of 3-HBA and albendazole may be a promising adjunct therapy for human angiostrongyliasis.
